# Players’ selection for basketball teams, through Performance Index Rating, using multiobjective evolutionary algorithms

**DOI:** 10.1371/journal.pone.0221258

**Published:** 2019-09-04

**Authors:** Miguel Ángel Pérez-Toledano, Francisco J. Rodriguez, Javier García-Rubio, Sergio José Ibañez

**Affiliations:** 1 Quercus Software Engineering Group, University of Extremadura, Cáceres, Spain; 2 Computer Science Department, University of Burgos, Burgos, Spain; 3 Group in Optimization of Training and Sports Performance, University of Extremadura, Cáceres, Spain; 4 Facultad de Educación, Universidad Autónoma de Chile, Santiago de Chile, Chile; University of Hong Kong, HONG KONG

## Abstract

In any sport the selection of players for a team is fundamental for its subsequent performance. Many factors condition the selection process from the characteristics of the sport discipline to financial limitations, including a long list of restrictions associated with the environment of the competitions in which the team takes part. All of this makes the process of selecting a roster of players very complex, as it is affected by multiple variables and in many cases marked by a great deal of subjectivity. The purpose of this article was to objectively select the players for a basketball team using an evolutionary algorithm, the Non-dominated Sorting Genetic Algorithm II (NSGA-II) that uses stochastic search methods based on the imitation of natural biological evolution. The sample was composed of the players from the teams competing in the top Spanish basketball league, the Association of Basketball Clubs (ACB). To assess the quality of the solutions obtained, the results were compared with the teams in the ACB playing in the same competition as the players used in the study. The results make it possible to obtain different solutions for composing teams rendering financial resources profitable and taking into account the restrictions of the competition and of each sport management.

## Introduction

The selection of players for a team in any sport is fundamental for its subsequent collective performance [1]. There are many factors that condition the selection of the players, from the characteristics of the sport discipline, the competitions in which they take part, the regulations of the team’s reference country, the tradition or philosophy of the club, the prior undertakings/contracts established by the club or player, to financial limitations, and the characteristics of the type of play the coach intends to develop. All of this makes the process of selecting a roster very complex as it is affected by multiple variables and often marked by a great deal of subjectivity. Every sport discipline needs a minimum number of players to compete and a basic roster to be able to train and perform during the whole regular season. Not all the players in the team play the same role in the match. It is necessary when building up the team to define the number of players required for each specific position. This choice will be conditioned by the philosophy that the coaching staff wants to apply and the financial resources available. The selection of the components of a team as a function of the roles to be developed is complex, even more so when the professionals have different performance characteristics [2]. Furthermore, depending on the role of each player, the competences that serve to analyze their performance are different.

In basketball, the teams can participate simultaneously in two or three competitions during a season. On the one hand they compete in the regular league [3] and on the other in an elimination tournament (Cup), and the best teams participate in an international competition against the best teams of the continent [4]. Sometimes the number of players that can participate in all these competitions is different, varying from 10 to 12. The contribution of these players to the performance of the team is not the same, and roles for first team players and substitutes are determined [5]. There are also limitations regarding players’ nationalities or ages. In addition it is necessary to know which performance indicators predict victory and performance in each sport. It has been almost unanimously determined in basketball that the indicators that predict victory in a match are the number of field baskets scored and defensive rebounds secured [3, 6], although these may be different depending on the competition. In the Spanish basketball second league, in 82.4% of the cases assists, steals and blocks make it possible to identify the best classified teams at the end of a season [7]. On the other hand, in the ACB league, assists, defensive rebounds and field goals were related with the winning teams in 86.7% of the games [8]. Each competition, according to situational variables, shows different performance indicators that determine wins or losses. Therefore, for the composition of a roster adapted to the characteristics of this professional competition, players were needed that had great passing skills and defensive intensity to provoke steals and blocks. Similarly, the different performance profiles presented by each playing role have been studied (point guards, forwards and centers) [5, 9]. For example, a center in the NBA American league does not need a good performance in 3 point shots, while in the Spanish ACB league it is an important factor to be taken into account [9]. There is general consensus on the use of easily objectifiable technical-tactical performance indicators, recorded by competition analysts and there are different formulas to calculate individual player performance based on these indicators [10–12]. The formula used by the ACB League (the analyzed competition) was the one chosen for the present study.

The specific rules of each competition establish the number of players that can be registered, the number of players that can be added to or taken off the roster, the reasons that a player can be changed, their original nationality, and occasionally quotas for players per age, to favor the progression of young talents. The conditions for contracting the players for each competition are reflected in the players’ collective bargaining agreements [13]. The sport directors charged with composing a roster of players have to take these restrictions into account before recruiting new players. Similarly, the club budget has a great deal of influence on the final performance in all sports in the short, medium and long term and directly affects the composition of the roster [14, 15]. For example, in English Premier League soccer, in the last decade, the teams that ended the season in the first four places in the classification were the ones that had spent the most money on the players’ salaries in those seasons [16].

The high number of players available in the market thanks to its liberalization and internationalization, the number of different leagues from which to select players and the ease with which some clubs obtain a generous budget to engage players make this selection increasingly difficult. It seems more and more necessary to apply objective methods for player selection that optimize the possibilities for choice as a function of the variables or performance indicators needed for each playing position [1, 2, 17].

The problem of obtaining a group of players in a team where each player has a different role, and at the same time trying to optimize two different objectives i.e. maximizing expected performance and minimizing contracting costs, can be approached in different ways. One approach is to formulate it as an optimization of one single objective with restrictions [18, 19]. For this purpose, an assessment function must be constructed to weight both objectives at the same time. Dynamic programming, genetic algorithms and branch and bound are some examples of techniques commonly used to solve this type of problems, obtaining a single solution as the result. This approach has several disadvantages. The main one is that it requires an adjustment of the assessment function that is difficult to perform, and can bias the search for a suitable solution [20, 21]. To improve the results of the previous approach the problem can be formulated as a multiobjective problem. As opposed to the former, multiobjective optimization obtains a set of solutions where the decomposition of the assessment function into different objectives leaves room for more flexible solutions that cannot be reached with the single objective approach [22]. This set of solutions makes it possible to assess each of the solutions obtained individually so that the sport coaching staff of each club can choose the most suitable one for its context and circumstances.

Researchers have previously analyzed players’ performance in different sports using a series of attributes associated with diverse aspects of each sport, with the purpose of being able to select players individually, like [23–26]. It has been shown that the selection of players by team managers does not only take into account the best individual performance indicators. In baseball, teams are composed using the Hungarian Method (HM). HM is designed to assign tasks to players of the team, so that their interactions improve the overall performance, but it is not designed to select players and include them in a roster. Moreover, HM only take into account the expected performance of the player but does not evaluate their cost. Yee et al. [27] showed that the combined use of the HM for forming baseball teams, using strategies that apply the Nash Equilibrium (NE) or Pareto Efficiency (PE) improved the team’s performance in matches. Despite this, few studies have focused on providing complete teams. In [28], an optimization model to compose a team of players for football clubs, with the objective of maximizing the sum of the transfer market appreciation of the players in the team, is presented. In addition, in [1] an EA NGSA-II is used for the selection of rosters in cricket in order to maximize batting, bowling, and field performance.

However, as far as we know, there is not any previous work dealing with the optimal selection of basketball teams. At this point, it is important to highlight that it is not possible to use previous approaches for the selection of players in other sports since composing a basketball team goes beyond selecting a certain number of players according to a particular performance measure. It is necessary, on the one hand, to ensure that the team is composed of a minimum number of players who are capable of playing in each of the usual positions or roles in this sport, i.e., point guards, forwards. etc. In addition, differences with others sports, such as American football, reside in the difficulty to find a unique metric to assess all the players’ performance. In American football, each team has special and different compositions for attack, defense and others special situations, with different players, roles, and functions. On the contrary, our method provides a unique valuation for any role, where all the players have to play in both phases of the game, attack and defense. Moreover, in some sports (mostly in the USA), players enter the league via draft: they are selected from a candidate pool in a pre-determined order [29], being therefore available depending on other teams decisions, which is not the case to compose a basketball team in the present study. Teams in the Spanish League can compose their roster with all the players available in the market and, due to the actual physical demands and congested fixture, all the players have to be ready to play, making the differences between starters and non-starters low.

In this study, we formulated the problem of the selection of basketball players to compose a team whose performance is maximized while its cost is minimized. For this purpose, we employed an EA NSGA-II [30, 31]. The performance data and cost of the players in the main Spanish competition, the ACB league, corresponding to the 2014-2015 season were used to run the algorithm, and to show the suitability of the rosters of players obtained. The results were compared with the performance of the teams that played in the ACB in the following season (2015-16). The development of this tool will permit the objective selection of players for team composition using objective and contrastable data that make it possible to select coherent and efficient rosters, taking into account the established restrictions (country of birth, age, budget, etc.) and satisfying the requirements of the technical staff.

The rest of the article is structured as follows. Firstly, we detail the material and methods employed in the article. Secondly, we show the experimental results obtained and perform a discussion about them. Finally, we draw the conclusions and future work.

## Materials and methods

### Sample

The players selected were those who played in the ACB League from 2014-2015 (n = 286). To assess the results of the study, they were compared with those of the 2015-16 season, as in these two seasons there were no promotions or demotions in the ACB League and the majority of the players played in the same competitions. If performance data had been used from players from other competitions, the study would have provided equally valid data but the comparative analysis of the results obtained would have been altered because of the inclusion of data collected from another context. Different information sources were used to calculate the financial cost of the players, from information published on the websites of the different teams, to public information broadcast by different media and the data from the collective bargaining agreement of the ACB League players. The data on the players were obtained using the information from the databases that recorded the performance indicators of the players in the rosters participating in the ACB League in the 2014-2015 season (http://www.acb.com/). The purpose was to find players whose valuations were obtained in the same context so as not to detract from the results of the study by using data on players from competitions with different levels of demand [9].

Game related metrics are used to measure player’s performance. In Europe, the *Performance Index Rate* (PIR) is the most widely used rating index and is commonly used by managers and trainers. In addition, the recruitment agencies use this index, since it allows the comparison of players from different leagues. The Performance Index Rating (PIR) is the mathematical model used by the International Basketball Federation (FIBA) to valuate players after a match, and includes different performance indicators all together. It is a part of the Tendex basketball rating system [10]. The Tendex method makes it possible to analyze the global performance of a player (Tendex Global), centering the valuation on the attack phase (Tendex Offensive), or on the defensive phase (Tendex Defensive), in a way which is similar to the NBA’s Efficiency (EFF) stat. Other methods for quantifying basketball players’ performance use similar indicators as those used in the PIR method, but weight each of the actions in the final valuation, like Tendex [10] or Four Factors [11]. However all these metrics for valuating players have the same conceptual limitations for the selection of a roster of players, as these indicators do not provide qualitative information on players’ behavior. The real performance of a team is not just the sum of the player’s performance, because interactions among players are important. In a basketball game there are intangible variables that are not measurable, such as blocks or defences, which are decisive for the performance of the team. For this reason, the values obtained in this work must be completed by coaches who evaluate the intangible variables that are not collected in this study.

The PIR is currently used in the main European international competitions like the EuroLeague and the EuroCup, as well as various European national domestic and regional leagues. It is also used in these competitions to reward the most valuable player (MVP). PIR has also been used as a reference indicator in several previous works [32–36]. In addition, to compare all players’ performance, PIR were normalized according to games played. The homogenization of the data made it possible to compare the players who played during the whole competition and showed a better performance with those who only played some matches.

The information on the players and their performance was obtained from public sources freely available for everyone. Nevertheless players’ names have been omitted for the sake of data protection and privacy. Performance indicators of the players and teams included in this study are available to the public as they are published on the League web pages and those of the teams participating in the competition. Similarly the financial data used in this study, that conditions the distribution of the teams’ budgets, was obtained from the League web site, from the teams themselves and from the information published in the different media when a player is engaged for a team. In some cases estimates of the players’ salaries were made based on the collective bargaining agreement in force in the ACB League.

### Procedure

In this section, we describe in detail the algorithm proposed to deal with the selection of basketball players, maximizing team performance and minimizing its cost. Team performance was estimated by summing players’ scores assigned by the ACB league. Therefore, the aim was to select a subgroup of players to improve performance while minimizing its cost and taking into account the restrictions detailed in this section. It is important to note that other features could be chosen to estimate team performance without any changes in the proposed algorithm. It is important to highlight one of the main advantages of the tool, currently the number of players available in the market is increasing highly. Therefore, this type of tool allows a pre-selection of high-performing teams according to a certain evaluation measure (in this case PIR, but others might be used), so that in comparison the work to be done in order to analyze the proposed teams by the coach or the manager must be much smaller. Moreover, it would be easy to generate partial rosters by just adjusting the maximum number of players in each position, which will allow us to complete existing teams.

The EA used in this study (NSGA-II) [31], considers two opposite objectives. On the one hand to minimize the financial cost of building the roster and on the other to maximize the expected performance of the players selected. The valuation index used since 1991 in the ACB League was utilized to analyze player performance. It is also a reference indicator of the most valued players (MVP) in the majority of important competitions played in Europe and has been used widely in scientific research [32–34]. It is calculated with the following formula:

$$\begin{array}{c} \begin{array}{cc} Valuation= & (Points+Rebounds+Assists+Steals+Blocks+Fouls\;received)-\\ & (Unsuccessful\;field\;shots+Unsuccessful\;free\;shots+\\ & Blocks\;received+Losses+Fouls\;committed) \end{array} \end{array}$$ (1)

The team valuation index is the sum of all players’ normalized valuation indexes, and the valuation of the teams and players using this formula is employed by team managers and coaches to identify the most complete players. Thus it can be said that it is an objective performance indicator which is widely accepted in the sport context. It also serves to identify the team’s performance during the competition. For example in this study a simple regression analysis was used to predict the importance of the team valuation in the final classification. In this study, valuation is a simple index that explains 46% of the variation in the final ranking for the ACB competition in the respective season (Table 1).

**Table 1:** Effects of team’s valuation index on final ranking in the ACB league. Season 2014-2015. Sample size: 340 matches. Dependent variable: ranking; Predictor variable: Valuation index.

	*R*^2^	Adjusted *R*^2^	Sig.
Model 1	0.493	0.462	.001

### Restrictions

The restrictions associated with the execution of the algorithm proposed in this article were as follows: i) the cost of the rosters of the teams formed had to have a maximum limit of 3 million euros. The data associated with this season were used to analyze the teams and players; ii) the number of players per team was 12, with players in 5 different roles. Each team had to be composed of 3 point guards, 2 shooting guards, 2 forwards, 3 power forwards and 2 centers. These data are similar to the composition of the team rosters in the ACB League. It should be pointed out that, according to the performance indicators commented on above, some players may play in different positions and thus be candidates for several positions, having the same PIR valuation for all the positions (two positions at most in this work); iii) the competitive system in the ACB League limits the number of players according to their country of origin and their training. Thus there can only be a maximum of 2 players from outside the European Union (EXT). There can be a maximum of 1 player who was born in a former Spanish colony (COT). There can be up to 5 players born in, or with a passport from, a European Union country (EUR) other than Spain; and lastly there must be at least 4 players born in Spain or trained up to the age of 18 in Spanish teams with the possibility of going into the national team (JFL); iv) with these conditions, in this study executions were run which offered a set of solutions that was limited to 100 teams. For this simulation complete teams were generated. When developing the algorithm new restrictions can be included like players with current contracts or a smaller number of players if the team has given undertakings to other players. If the algorithm was executed again a set of different solutions would be generated, as EAs are stochastic search methods based on imitating biological evolution in nature. The set of solutions obtained is formed by a set of complete “non-dominant” rosters, that is to say that none of the rosters obtained is better than the others in all the objectives. Thus it is necessary for the sport management of the clubs to choose the solution that is best adapted to their interests.

**Fig 1:**
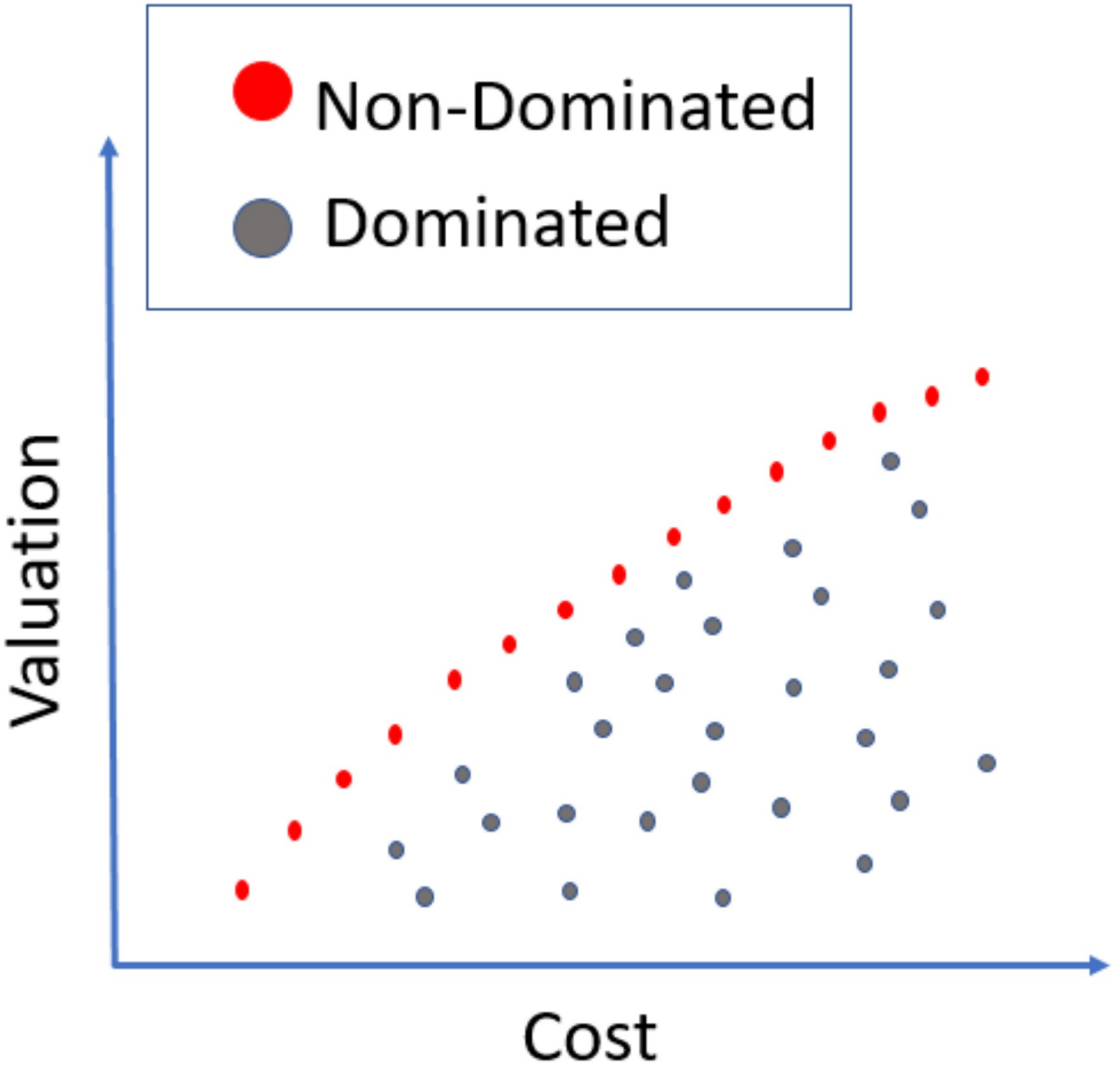
Pareto front example.

It is important to note that there is not a unique solution for multiobjective problems but a set of non-dominated solutions, that is, those for which there is not another feasible solution better in all objective functions. Fig 1 shows an example of a Pareto front. Determining exactly the Pareto front for multiobjective combinatorial optimization problems is quite difficult. There exist few exact methods to determine the Pareto front and we can expect to apply these methods only for very small instances [37]. But the greatest interest is in solving this problem for large instances involving very large numbers of players. As indicated previously, nowadays the number of players available in the market is highly increasing thanks to its liberalization and internationalization.

It is precisely in this type of situations where the application of metaheuristics has shown greater effectiveness and has become a common tool [37]. Solving these kinds of problems is not a trivial task [38]. For these reasons, finding the Pareto front is not practical in general, so the goal becomes to get an approximation of the Pareto front by using non-exact algorithms. In this context, the most widely used techniques are metaheuristics, a broad family of solvers including EAs, swarm intelligence algorithms, and many others.

EAs are stochastic search methods based on mimicking natural biological evolution [39, 40]. EAs employ a population of individuals that represents points in the solution space of a given problem. Those individuals (basketball teams) are then evolved by means of probabilistic operators such as mutation, selection, and (sometimes) recombination to obtain increasingly better individuals, that is, better solutions for the problem at hand. EAs have shown outstanding performance when facing difficult optimization problems due to their ability to locate the most interesting regions of vast and complex search spaces. In particular, in the last decade, EAs have become the most extended tool to solve real-world multiobjective problems [41]. Multiobjective evolutionary algorithms basically add to traditional EA methods to promote solution diversity. It is important to note that there is not a unique solution for multiobjective problems but a set of non-dominated solutions, that is, those for which there is not another feasible solution better in all objective functions. Fig 1 shows an example of a Pareto front.

A wide variety of multiobjective evolutionary algorithms was proposed in the literature [42]; the NSGA-III [43], MOEA/D [44], and SMS-EMOA [45] to mention but a few. Among the vast amount of proposals, we have chosen the NSGA-II, a previous version of the NSGA-III, since the latter is intended for problems with more than three objectives. Moreover, the NSGA-II stands out as one of the most widely used proposals [46] and is nowadays the tool of choice to deal with many real-world multiobjective optimization problems [47, 48]. We detail below the basic NSGA-II scheme, emphasizing the methods that have been modified to adapt it to the problem in question. In particular, we describe solution representation, initial population generation and genetic operators, that is, crossover and mutation.

### Problem formulation

The problem stated above can be formulated in a formal manner as follows:

$$\begin{array}{cc} \text{Minimize:}\; & \sum_{i=i}^{n}C_{i}x_{i} \end{array}$$ (2)

$$\begin{array}{cc} \text{Maximize:}\; & \sum_{i=i}^{n}P_{i}x_{i} \end{array}$$ (3)

$$\begin{array}{cc} \text{Subject}\;\text{to:}\; & \sum_{i=1}^{n}x_{i}=TS \end{array}$$ (4)

$$\begin{array}{c} \sum_{i=1}^{n}R_{i1}x_{i}\;\geq \;PG \end{array}$$ (5)

$$\begin{array}{cc} & \sum_{i=1}^{n}R_{i2}x_{i}\;\geq \;SG \end{array}$$ (6)

$$\begin{array}{cc} & \sum_{i=1}^{n}R_{i3}x_{i}\;\geq \;F \end{array}$$ (7)

$$\begin{array}{cc} & \sum_{i=1}^{n}R_{i4}x_{i}\;\geq \;PF \end{array}$$ (8)

$$\begin{array}{cc} & \sum_{i=1}^{n}R_{i5}x_{i}\;\geq \;C \end{array}$$ (9)

$$\begin{array}{cc} & \sum_{i=1}^{n}N_{i1}x_{i}\;\leq \;EX \end{array}$$ (10)

$$\begin{array}{cc} & \sum_{i=1}^{n}N_{i2}x_{i}\;\leq \;CO \end{array}$$ (11)

$$\begin{array}{cc} & \sum_{i=1}^{n}N_{i3}x_{i}\;\leq \;EU \end{array}$$ (12)

$$\begin{array}{cc} & \sum_{i=1}^{n}N_{i4}x_{i}\;\geq \;JF \end{array}$$ (13)

$$\begin{array}{cc} & x_{i}\in \left\{0,1\right\}\;\forall i=1,\ldots ,n \end{array}$$ (14)

where *x*_*i*_ = 1 if the player *i* is selected for the team and 0 on the contrary. The objective is to minimize the cost (Eq 2 ) and maximize performance (Eq 3) taking into account the cost *C*_*i*_ and valuation *P*_*i*_ of each player (as indicated in the Procedure Section) by selecting a team of *TS* players (Eq 4) from a set of *n* available players. Eqs 5–9 state the number of players in each position, that is, *PG* point guards (Eq 5), *SG* shooting guards (Eq 6), *F* forwards (Eq 7), *PF* power forwards (Eq 8) and *C* centers (Eq 9). Each player is previously assigned at most two roles where he can play. In order to represent that information in the mathematical model, we create a matrix *R* so that if player *i* can play the role represented in the position *j* then *R*_*ij*_ = 1, 0 on the contrary. In particular, *j* = 1 stores information for point guards, *j* = 2 for shooting guards, *j* = 3 for forwards, *j* = 4 for power forwards, and *j* = 5 for centers. Note that according to the problem’s requirements the number of players in each position must be equal to *PG*, *SG*, *F*, *PF*, or *C*, respectively, but might be higher since some players can play in two different positions. In a similar way, the number of players in a team according to their country of origin and training is established by Eqs 10–13. In particular, Eq 10 sets a maximum of *EX* EXT players, Eq 11 a maximum of *CO* COT player, Eq 12 a maximum of *EU* EUR players, and Eq 13 sets the minimum of JFL players in *JF*. In order to store nationality information for each player, we use the matrix N where if the player belongs to the region indicated by *j* then *N*_*ij*_ = 1, 0 on the contrary. Specifically, *j* = 1 stores information for players from outside of the European Union (EXT), *j* = 2 for players born in a former Spanish colony (COT), *j* = 3 for players with passport from a European Union country different from Spain (EUR), and *j* = 4 for players born in Spain (JFL). According to the restrictions detailed in the above section, the particular values for the different constants in the model are summarized in Table 2.

**Table 2:** Values for the constants in the problem formulation.

Constant Name	Description	Value
TS	Team Size	12
PG	Number of Point Guards	3
SG	Number of Shooting Guards	2
F	Number of Forwards	2
PF	Number of Power Forwards	3
C	Number of Centers	2
EX	Number of EXT Players	2
CO	Number of COT Players	1
EU	Number of EU Players	5
JF	Number of JFL Players	4

#### NSGA-II basic scheme

The NSGA-II basis scheme is depicted in Algorithm 1. It starts by initializing a population of random solutions (line 1) that are evaluated according to the cost (Eq 2) and valuation (Eq 3 ) of the basketball teams represented by those solutions (line 2). The NSGA-II proposes a method based on sorting the population into a hierarchy of sub-populations using Pareto dominance criteria (function FastNonDominatedSort (line 3). Then, solutions are selected according to the mentioned hierarchy (line 4) and crossover and mutation operators are applied (line 5). This process is repeated (lines 7-23) until a predefined stop condition is reached (line 6). In order to promote diversity in the Pareto front, solutions are also ordered taking into account the similarity between members of each sub-group (function CrowdingDistanceAssignment, line 13). A more detailed description of these functions can be found in the original paper [31]

**Algorithm 1:** NSGA-II basic scheme.

 **Input:** *PopulationSize*, *P*_*crossover*_, *P*_*mutation*_

 **Output:** *Children*

**1** *Population* ← *InitializePopulation*(*PopulationSize*)

**2** *Evaluate*(*Population*)

**3** *FastNondominatedSort*(*Population*)

**4** *Selected* ← *SelectParentsByRank*(*Population*)

**5** *Children* ← *CrossoverAndMutation*(*Selected*, *P*_*crossover*_, *P*_*mutation*_, *P*_*mutation*_)

**6 while** not *StopCondition*() **do**

**7**  *Evaluate*(*Children*)

**8**  *Union* ← *Merge*(*Population*, *Children*)

**9**  *Fronts* ← *FastNondominatedSort*(*Union*)

**10**  *Parents* ← ∅

**11**  *LFront* ← 0

**12**  **for** *Front*_*i*_ ∈ *Fronts* **do**

**13**   *CrowdingDistanceAssignment*(*Front*_*i*_)

**14**   **if** *Size*(*Front*_*i*_)+ *Size*(*Parents*)< = *Size*(*Population*) **then**

**15**    *Parents* ← *Merge*(*Parents*, *Front*_*i*_)

**16**   **else**

**17**    *LFront* ← *i*

**18**    Break()

**19**   **end**

**20**  **end**

**21**  **if** *Size*(*Parents*)<*Size*(*Population*) **then**

**22**   *SortByRankAndDistance*(*Front*_*LFront*_)

**23**   *Fill*(*Parents*, *Front*_*LFront*_)

**24**  **end**

**25**  *Selected* ← *SelectParentsByRankAndDistance*(*Parents*)

**26**  *Population* ← *Children*

**27**  *Children* ← *CrossoverAndMutation*(*Population*, *P*_*crossover*_, *P*_*mutation*_)

**28 end**

#### Solution representation and population initialization

**Fig 2:**
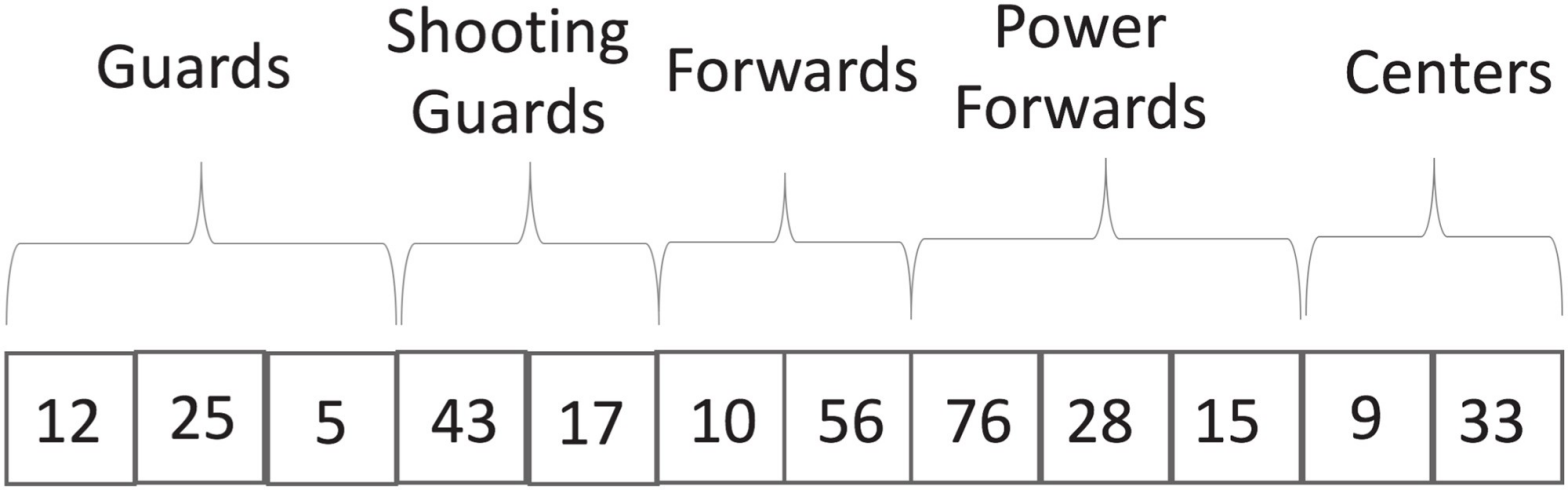
Solution representation.

A representation scheme has been used in which each team is represented by a vector of size n, where n stands for the number of players allotted in the team. In that case, we contemplate teams of a fixed size, which is 12 (Eq 4 ). Therefore, each position in the vector stores an integer that uniquely identifies a player. Moreover, the position in the vector also represents the role of the corresponding player in the team. Players in the three first positions play the point guard role on court (Eq 5), the next two players play the shooting guard role (Eq 6), the next two the forward role (Eq 7), the next three the power-forward role (Eq 8), and the last two players play the center role (Eq 9). Fig 2 shows an example of the representation used, in which, to mention but a few, player number 12 plays the role of guard, player 43 shooting guard, and player 9 center.

In order to generate a whole population of individuals, we have devised a procedure to create *PopulationSize* initial solutions. This procedure just assigns a random player not selected previously, taking into account the role of the current vector position. It is important to note that the representation employed helps the algorithm to guarantee solution feasibility. By using a vector of size 12, we are generating teams with exactly 12 players. Moreover, by associating roles to some positions in the vector, the procedure to generate initial solutions only proposes players that play a particular role when selecting them for a position. With regard to the constraint related to the player’s country of origin, the solution initialization procedure counts the number of players per origin, excluding players from those origins for which the maximum number of players has been reached (Eqs 10–13).

### Crossover and mutation operators

**Fig 3:**
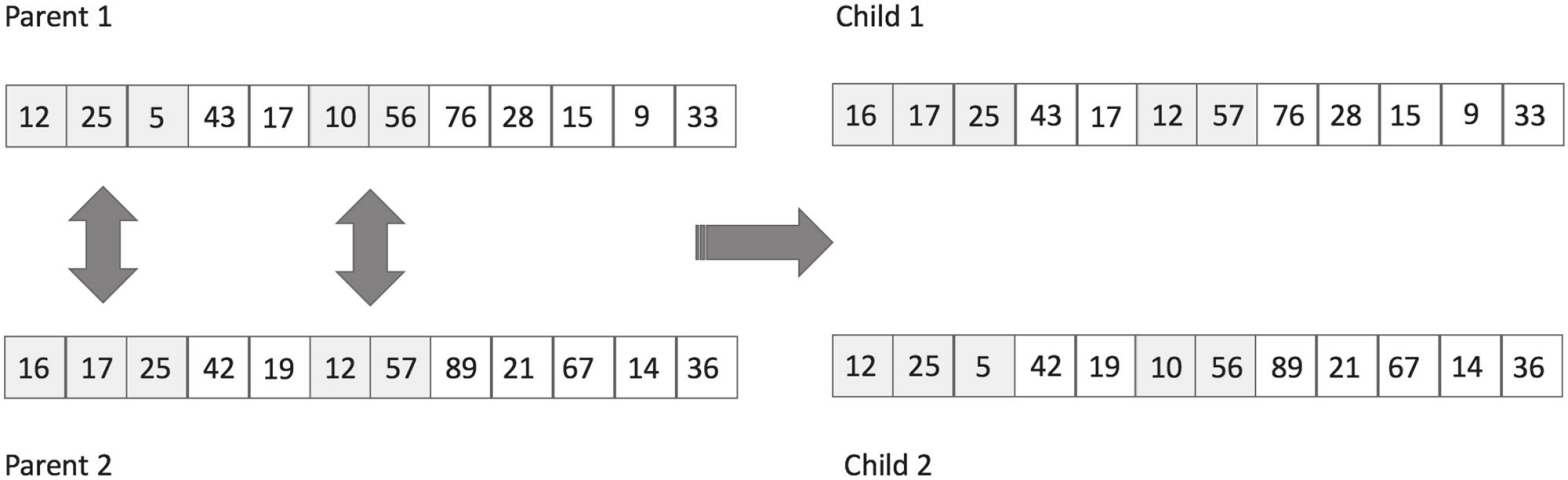
Crossover operator.

The crossover operator combines information on two or more teams from the current population to generate new teams. For this purpose, we accordingly selected two parents and interchanged the players of two different roles chosen at random, generating two new teams. To do this, the values of each position of the vector belonging to the selected roles were exchanged. It is important to note that the crossover operator needs to generate feasible teams. Therefore, individual exchanges are only made if the new player was not previously in the team and the constraint of the number of players per origin is not breached (Eqs 10–13). Fig 3 presents an example of a crossover operation in which the interchanged roles are guards and forwards.

The mutation operator usually modifies a chosen team randomly. Following this premise, the mutation process selects a new player for the chosen team, taking into account the role of the player and checking that the new player was not previously assigned to the team in order to maintain feasibility. Moreover, the new player is selected from those origins for which the maximum has not yet been reached (Eqs 10–13).

## Results and discussion

The execution of the algorithm, commented in the previous section, was configured so that it generated teams of 12 players with a maximum cost of 3 million euros, and a set of maximum solutions of 100 different teams. Given the volume of the results obtained in the different simulations performed in this study, these results are available for consultation in the S1 Appendix. Similarly the values referring to the statistical data on the players and the costs of the teams used in the study, obtained from the ACB webpage, are also available for consultation in the S2 Appendix. However, we have included in Table 3 a summary indicating the number of players, averaged valuation per minute, and averaged cost for each position.

**Table 3:** Number of players, averaged valuation per minute and averaged cost for each position.

Position	Number of Players	Averaged Cost	Averaged Valuation
Point Guards	71	288098.59 €	5.04
Shooting Guards	78	331217.95 €	6.65
Forwards	69	260072.46 €	5.94
Power Forwards	95	296789.47 €	4.43
Centers	49	358673.47 €	9.5

**Fig 4:**
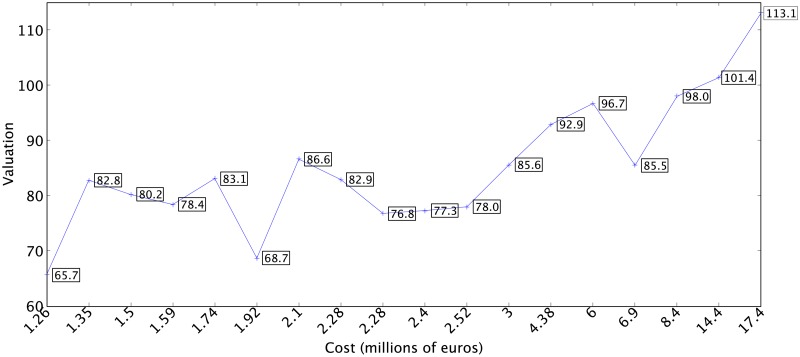
Costs of the rosters of the ACB teams in the 2015-2016 season.

In order to show that the method is able to generate cost-efficient teams using only past data, the quality of the simulations obtained was compared with the results of the cost and valuations obtained for the teams that played in the ACB League during the following season, 2015-2016. Fig 4 shows the costs of the rosters of the ACB teams in the 2015-2016 season. A positive trend can be seen connecting the cost with the valuations obtained by the teams in this season [14, 49]. The increase in the budget does not guarantee an automatic increase in the expected performance, as the money spent on the roster does not predict the team’s exact position in the final classification [14]. Knowledge of previous performance is of vital importance when constructing new rosters capable of generating collective behavior that make it possible to achieve success [14].

**Fig 5:**
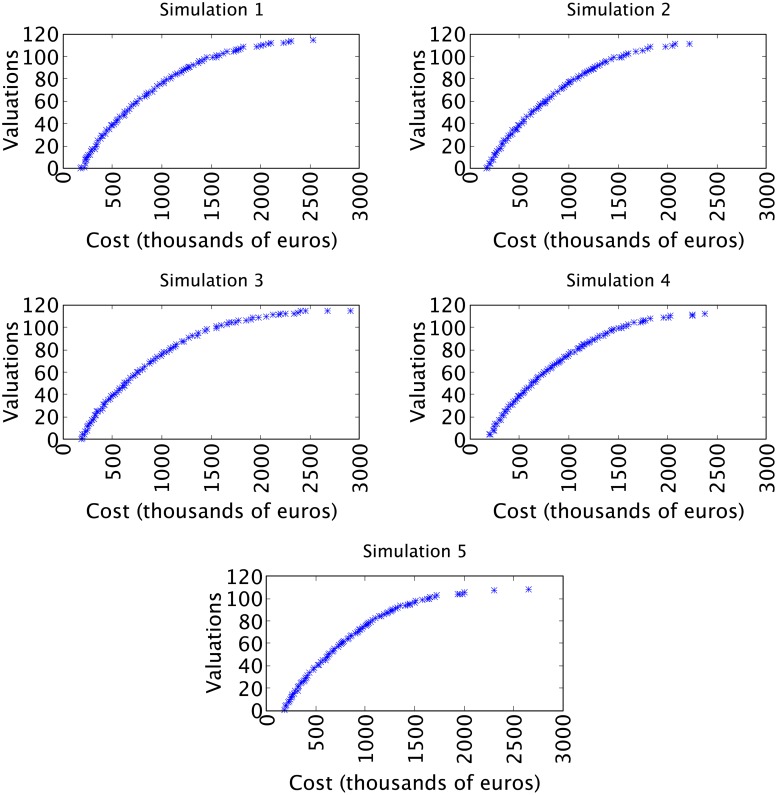
Simulations obtained using the NSGA-II algorithm.

Fig 5 shows a set of simulations, each of which has generated a front of solutions obtained using the NSGA-II constructing rosters of players. Each of the simulations has created 100 teams, at a cost of between 180,000 euros and 3 million euros, and valuations than varied from 0.44 to 115.21. Between both limits there are rosters that increase the cost as the valuation grows. The objective of establishing a maximum of 3 million euros in the algorithm was to confirm that with a modest budget it was possible to obtain competitive rosters of players. It is also evident that all the simulations performed have the same tendency, so it can be affirmed that they behave in a similar fashion, independently of the initial roster of players randomly generated by the algorithm.

**Fig 6:**
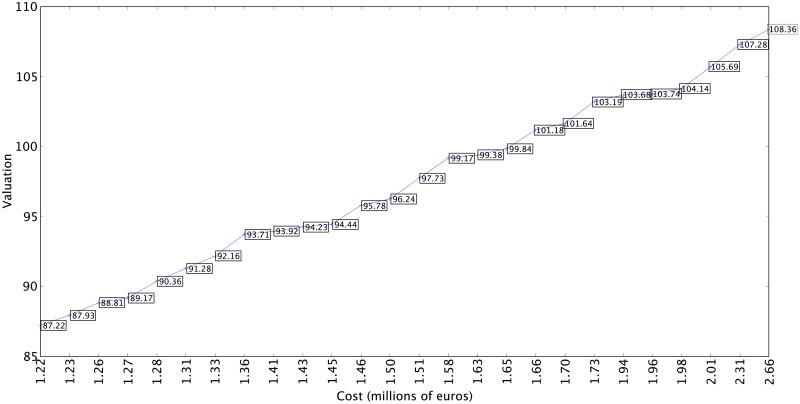
Fifth simulation with values from 1.2 million euros.

In the analysis of the results it would be advisable to only take into account the solutions with costs between 1.2 million (a minimum cost devoted to constructing a roster for the ACB League, according to the collective bargaining agreement) and 3 million, given that the ACB regulations demand a minimum investment per team. Furthermore, the majority of the teams generated with a budget of less than 1.2 million did not obtain competitive valuation results. Given that the simulations obtain similar results, the data from any of them can be analyzed randomly. To better visualize the results, and facilitate the comparison, Fig 6 shows the numerical data from some valuation information obtained from the Pareto front resulting from simulation 5. This graph shows that valuations of 104.14 are achieved at a cost of less than 2 million (1,980,000 euros). A study of the valuation and cost of the teams in the 2015-2016 season presented in Fig 4 shows that only one team with a cost of 17.4 million euros was capable of attaining higher scores. Furthermore, with budgets of over 1,215,000 euros minimal valuations of 87.22 are obtained which, compared with the cost of the teams in Fig 4, are only achieved with much higher budgets. Moreover, the results are not sporadic cases; any one of the simulations shows that the selection of players using the evolutionary algorithm obtains very competitive results.

Tables 4–6 show three different solutions to the problem being considered. For each roster, we detail the identification number of each player (*Player Id*) which corresponds to the number by which that player can be identified in S1 and S2 Appendices, his condition according to his nationality and training (*Contract type*), his cost (*Cost*), valuation averaged per minute played (*Val. Min*.), age (*Age*), and his main and secondary role (*Role 1* and *Role 2*, respectively). For the sake of simplicity, roles are specified with a numeric code, being 1 guards, 2 shooting guards, 3 forwards, 4 power forwards, and 5 centers. Number 0 is specified for the secondary role in case no secondary role is assigned to the corresponding player. Three different rosters have been constructed as a function of the budget in simulation number 5. There is a roster corresponding to the lower limit (team 52), another to the upper limit (team 3) and an intermediate roster (team 70). It can be seen how the preferred solutions repeat several players in the rosters, as these present an acceptable valuation price relation. It is also evident that several players are “jokers”, that is they can play more than one role in the team.

**Table 4:** First example of roster with valuation 87.22 and cost 1,215,000.

Team: 52	Player ID	Contract Type	Cost	Val. Min.	Age	Role	Role
Valuation: 87.22 Cost 1,215,000	184	EUR	100000	4.54	30	1	2
	73	EXT	100000	9.3	33	1	0
	236	JFL	80000	4.48	25	1	0
	182	JFL	100000	6.44	34	2	0
	14	JFL	10000	0.0	21	2	0
	75	JFL	80000	9.66	24	3	0
	105	JFL	125000	7.31	30	3	0
	32	JFL	150000	12.02	25	4	0
	142	EUR	70000	7.46	29	4	0
	104	EXT	150000	10.92	28	4	0
	74	JFL	150000	7.61	36	5	4
	165	COT	100000	7.48	34	5	0

**Table 5:** Second example of roster with valuation 90.84 and cost 1,650,000.

Team: 70	Player ID	Contract Type	Cost	Val. Min.	Age	Role 1	Role 2
Valuation: 99.84 Cost: 1,650,000	106	EUR	150000	6.49	31	2	1
	73	EXT	100000	9.3	33	1	0
	30	JFL	300000	9.94	29	1	0
	182	JFL	100000	6.44	34	2	0
	60	JFL	250000	8.56	36	2	3
	75	JFL	80000	9.66	24	3	0
	105	JFL	125000	7.31	30	3	0
	32	JFL	150000	12.02	25	4	0
	142	EUR	70000	7.46	29	4	0
	104	EXT	150000	10.92	28	4	0
	146	EUR	75000	4.26	33	5	0
	165	COT	100000	7.48	34	5	0

**Table 6:** Third example of roster with valuation 108.36 and cost 2,655,000.

Team: 3	Player ID	Contract Type	Cost	Val. Min.	Age	Role 1	Role 2
Valuation: 108.36 Cost: 2,655,000	106	EUR	150000	6.49	31	2	1
	73	EXT	100000	9.3	33	1	0
	30	JFL	300000	9.94	29	1	0
	119	JFL	400000	8.03	34	3	2
	60	JFL	250000	8.56	36	2	3
	75	JFL	80000	9.66	24	3	0
	105	JFL	125000	7.31	30	3	0
	32	JFL	150000	12.02	25	4	0
	260	EUR	350000	9.96	26	4	5
	104	EXT	150000	10.92	28	4	0
	19	EUR	500000	8.69	28	5	4
	165	COT	100000	7.48	34	5	0

The results obtained permit the optimization of decision making in the selection of players on the basis of a series of previously established restrictions [1, 17]. The teams found using this principle have a theoretically high level of performance at a low cost in comparison with the teams playing in the ACB League the following season. The multiobjective algorithm can be applied to other sports where the players are clearly differentiated by their specific position and play different roles in the team, thus permitting the roles to be covered by the most capable players and meaning that there are no players that play several roles and therefore are overexploited [2].

**Fig 7:**
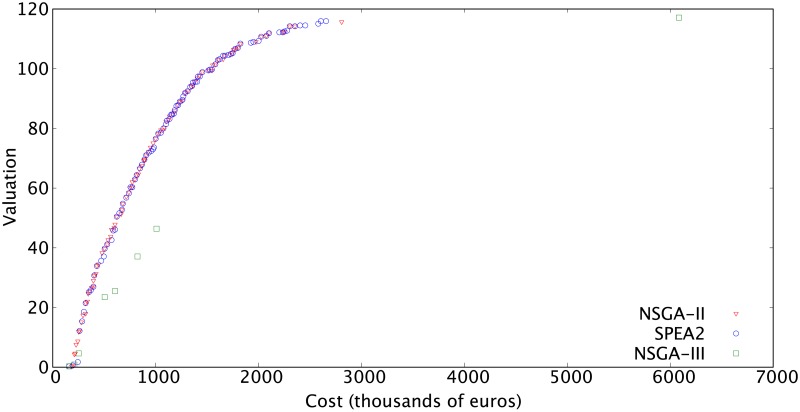
Approximation of the Pareto front obtained by NSGA-II, NSGA-III, and SPEA2.

Finally, in order to assess the performance of NSGA-II with respect to other widely used multiobjective evolutionary algorithms, we compare the results of our proposal versus SPEA2 [50] and NSGA-III [43]. Fig 7 shows the approximation of the Pareto front found by each algorithm. The results of NSGA-II and SPEA2 are quite similar, reaching solutions of comparable quality. It is important to take into account that SPEA2 and NSGA-III use the same problem representation and crossover and mutation operators that NSGA-II. We specifically designed this representation and these operators to deal with the particular features of the problem at hand. On the contrary, NSGA-III results are worse, despite employing the same representation and operators. The cost for a roster with the same valuation that NSGA-II or SPEA2 is much higher, increasing that difference as we want to get rosters with better valuations. At this point, it is worth mentioning that NSGA-III is specifically designed for problems with more than two objectives, which might explain its inferior performance.

## Conclusions

The use of the genetic algorithm NSGA-II will facilitate the construction of new rosters in basketball teams for the coaches and directors, minimizing cost and maximizing performance. Decision making is always a complex question, conditioned by different factors that affect the result of the selection. Therefore, the lower the level of uncertainty in this process the better the decisions that are made. However, this study does not aim to substitute the work done by the technical sport staff, but rather is meant to facilitate decision making in the selection of players eliminating subjectivity as far as possible. However, it is necessary to contextualize the results obtained and indicate some limitations of the study. Firstly, the group of players used presupposed that all of them were available to be integrated into the team, without taking into account that many of them may have had contracts that were still in force. Furthermore, the players selected were the ones with competitive valuations and low cost. It is probable that after carrying out a good season the cost of contracting many of them would have gone up. The algorithm considered in this study can be easily adapted to limit the selection of players solely for those positions where they are needed. In this line of thought, the algorithm can be adapted to use other different performance indicators as it is not necessary to focus solely on the valuations of the ACB League. Finally, the data used in the simulations obtain the expected evaluations of the teams the following year. However, it is not possible to guarantee that the performance of the players will remain the same the following year.

Moreover, the performance of a team is not the partial sum of the individual performance of the players. Thus the selection of players for making up a roster, based on the PIR method, or any other selection method, using evolutionary genetic algorithms, is only an aid in the coach’s decision making about player recruitment. Bearing in mind the different proposals of players, the coaches will collect information on the players’ behavior, and study their compatibility with the others, and the possible synergies and incompatibilities in play. From this starting point they will select the players who can best adapt to the coach’s game philosophy making it possible to improve their teammates and enhancing the team’s performance.

For future research new restrictions can be added to the roles of the players, like for example, point guards that generate a minimal number of assists, centers that achieve a minimal number of rebounds or small forwards that guarantee a number of points per match. New objectives can be added like minimizing the mean age of the players who form the roster, maximizing the number of years of experience in a determined competition, or selecting players with the aim of attracting fans from new markets.

Future possible lines of research, which would complete the results obtained in this study, could include carrying out a comparative study of how the results of the algorithm would be affected by using different valuation methods of player performance, and analyzing the use of new algorithms implementing metaheuristics that would make it possible to improve the results obtained with the NSGA-II.

## Supporting information

S1 Appendix (XLSX)

S2 Appendix (ZIP)
